# Clinical Holistic Medicine: Chronic Pain in Internal Organs

**DOI:** 10.1100/tsw.2005.27

**Published:** 2005-03-19

**Authors:** Søren Ventegodt, Joav Merrick

**Affiliations:** ^1^ The Quality of Life Research Center Teglgårdstræde 4-8, DK-1452, Copenhagen K, Denmark and The Scandinavian Foundation for Holistic Medicine, Sandvika, Norway; ^2^ National Institute of Child Health and Human Development, Office of the Medical Director, Division for Mental Retardation, Ministry of Social Affairs, Jerusalem and Zusman Child Development Center, Divisions of Pediatrics and Community Health, Ben Gurion University, Beer-Sheva, Israel

**Keywords:** quality of life, QOL, philosophy, human development, holistic medicine, public health, holistic health, holistic process theory, life mission theory, chronic pain, pain in internal organs, Denmark

## Abstract

Holistic medicine seems to be efficient in the treatment of chronic pain in internal organs, especially when the pain has no known cause. It is quite surprising that while chronic pain can be one of the toughest challenges in the biomedical clinic, it is often one of the simplest things to alleviate in the holistic clinic. These pains are regarded as being caused by repressed emotions and are explained as psychosomatic reactions. Using holistic medicine, the patients can often be cured of their suffering when they assume responsibility for the repressed feelings. The holistic process theory of healing states that the return to the natural (pain free) state of being is possible whenever the person obtains the resources needed for existential healing. This shift is explained by the related quality of life and life mission theories. The resources needed are “holding” or genuine care in the dimensions of awareness, respect, care, acknowledgment, and acceptance with support and processing in the dimensions of feeling, understanding, and letting go of negative attitudes and beliefs. The preconditions for the holistic healing to take place are “love” and trust. Obtaining the full trust of the patient, therefore, seems to be the biggest challenge of holistic medicine, especially when dealing with a patient in pain.

## INTRODUCTION

About 1 in 20 Danes suffers from recurrent or chronic pain in his or her internal organs[1]. If we use the example of chronic pelvic pain[2], it can result from a variety of abdominal and pelvic causes including endometriosis, pelvic inflammatory disease, adhesions, urogenital causes, and bladder complaints including overactive bladder, urinary tract infection, and interstitial cystitis (IC). Often, there seems to be no medical explanation for the pain — apparently there is no ill health that can be detected within the internal organs (i.e., the stomach, intestines, gall bladder, pancreas, liver, bladder, kidneys, or reproductive organs). In spite of numerous in-depth medical examinations, nothing is revealed. Nevertheless, the pain (such as primary vulvodynia, for example[3]) continues (even for years) resulting in severe disability for the patient.

To our knowledge and experience, even analgesics such as morphine have little effect on chronic pain. Many patients have surgery on the suspicion that the problems may have a hidden, structural cause, which sometimes turns out to be the case, especially in acute pain. However, the operation may also result in adhesions and other sequelae that might even aggravate the patient's pain. Sometimes, the patients have repeated operations that may even take place at the patient's instigation in the hope of sudden freedom from pain.

If pain is of a chronic nature, it is our impression that exploratory surgery rarely produces significant findings and that it has little effect on the pain. Often, no physical reason can be detected for chronic pain in internal organs, leading us to assume that the pain is psychosomatic. Psychosomatic pain is often randomly distributed in the body and cannot be located within specific physical structures, tissues, or organs. You believe that you have located it when pressing on a certain point, but the next moment it is gone. It is as if the obstruction behind the pain is living its own life in the body. The pain is a warning sign of something that has been repressed and something in the life of the patient is not as it ought to be. This type of pain does not go away until the patient understands what he or she needs to "learn from the pain". The problems will persist until the patient takes up the challenge, begins investigating what is going on (or in popular terms, the body is trying to tell you something), learns the lesson, and takes proper steps to amend it.

Part of the underlying, emotional pain that the patient is unwittingly trying to avoid by somatizing the pain lies in the acknowledgment that he or she has a personality imbalance due to personal flaws and weaknesses. Once patients reach this acknowledgment of their health status, they may soon achieve pain relief. When the patients understand the problem, they can also find a way to solve it. Then it is mostly just a question of time before they can move on. As simple as it might seem when expressed in this way, the process of supporting the patient and helping him or her go through the emotional pain and "take learning" is quite multidimensional. Let us take a brief look at some of the dimensions in a short review of our recent research in the field of "quality of life as medicine".

## CLINICAL HOLISTIC MEDICINE

The life mission theory[4,5,6,7,8,9] states that anyone has a purpose in life or a huge talent. Happiness comes from living this purpose and succeeding in expressing the core talent in life. To do this, it is important to develop as a person into what is known as "the natural condition". The "natural condition" accrues when the person knows himself and uses all his efforts to achieve his most important personal goals. The holistic process theory of healing[10,11,12,13] and the related quality of life theories[14,15,16] state that the return to the natural state of being is possible whenever the person gets the resources needed for existential healing. The resources needed are "holding" in the dimensions such as awareness, respect, care, acknowledgment, and acceptance with support and processing in dimensions such as emotion, understanding, and letting go of negative attitudes and beliefs. The precondition for the holistic healing to take place is trust and the intention for the healing to take place. Existential healing is not a local healing of any tissue, but a healing of the wholeness of the person, making him much more resourceful, loving, and knowledgeable of himself and of his own needs and wishes. In letting go of negative attitudes and beliefs, the person returns to a more responsible existential position and thus achieves improved quality of life. The philosophical change of the person healing is often a change towards facing and struggling with difficult problems and challenges instead of avoiding difficulties in life[17,18,19,20,21,22,23,24]. The person who becomes happier and more resourceful often becomes healthier, more talented, and more able to function[25,26,27].

## CASE STORIES

The treatment of pain in the internal organs begins with a comprehensive physical examination with the necessary tests to exclude somatic illness. According to the holistic process theory, the treatment involves a combination of body therapy, psychotherapy, and life philosophy exercises, where the patients are first supported in achieving a better understanding of their inner self by formulating a more positive attitude towards life and, finally, by living accordingly.

> **Female, aged 28 years, with psychosomatic abdominal complaints:** First visit: Diarrhoea for ten days with nausea; vomits every morning, complete loss of appetite, but drinks plenty. She was admitted to hospital by an emergency physician and referred for colonoscopy, numerous faecal cultures all turned out negative [faeces are stools; they are tested for amoebic abscesses, and parasites such as worms]. Current medication: Losec [omeprazole], Alopam [oxazepam], nausea-relieving suppositories of unknown brand. Something is wrong. I (first author) cannot immediately diagnose the source of the patient complaints. A second appointment for in-depth assessment is scheduled.
>
> Second visit: Abdominal problems persist. I (first author) detect that the patient is anxious due to the death of her aunt at the age of 31 years by stomach cancer. In the aunts case her disease was wrongly diagnosed as gall-bladder stones. So far the patient has been to the emergency medical services, where she was given large numbers of pills, including Losec – which did not help. She is determined to be admitted to hospital immediately and regrets the fact that she did not accept the offer to remain in the hospital until her assessment was complete. My decision is to readmit the patient, since the thought of waiting for a distant appointment is greatly distressing the patient.
>
> Third visit: when arriving at our third meeting she feels much better, now that the physical examinations in the hospital have shown her to be in good health. I postulate that her basic problem might be fear of dying, which leads to muscle tension, which causes abdominal and chest pain. We discuss her reasons for fear of dying, but the patient cannot concur. Further discussion into the matter unveils the amount of anxiety that disturbs the patient and she is advised to confront her fear instead of constantly avoiding it. At the end of the meeting the patient is diagnosed with anxiety neurosis. EXERCISE: She is then given an exercise to support her in confronting her anxiety and accommodating her feelings, when they overwhelm her.

The symptoms expressed by the patient looked like serious abdominal illness, but in fact diagnosed eventually as a simple somatization of anxiety. As the patient gathers better control, she will understand the essence of her suffering and might be prepared to confront and process her anxiety. After such a process takes place, the abdominal problems will disappear and be replaced by the underlying problem of which they were symptomatic. Once the anxiety is integrated, she will have learned something existential about life. This insight will strengthen her and will enable her to achieve a fuller life.

> **Female, aged 31 years, with abdominal pain and pain during intercourse:** The patient complains of continuous abdominal pain, which at times becomes severe. Pain is usually present during intercourse, especially during orgasm. The patient reveals that she suffered during previous relationship and suspects that the pain may be connected with her previous negative experience. Pelvic examination: normal. Smear taken. EXERCISE: The patient was instructed to write about her negative experiences during her previous relationships in as much detail as possible. When completing this task she was asked to read it aloud to a female friend or come for a second appointment to discuss her former experiences.

The patient showed many internal resources and needed little external help in order for her to solve her problems. The issue in this case was about getting the patient to change to a responsible and constructive perspective. Once the perspective is in place, the task is straightforward.

**Female, aged 39 years, with lower abdominal pain despite hysterectomy:** Medical history: She had lower abdominal surgery with removal of uterus and ovaries. After surgery pain was reduced, but problems persisted. There are accompanying sleeping problems. She often stays at home sick and not attending work. Her family physician has prescribed Pantoloc [pantoprazole] for chronic gastritis. She is also taking antidepressants. “I have many skeletons in the cupboard that I can’t or don’t want to remember,” she says, sadly, thinking of her childhood. Physical examination: Presents with chronic pain in the flanks that can be provoked by pressing on psoas muscles. Quality-of-life conversation: I (SV) explain the correlation between pain and a full “internal waste bin” and we agree to try gestalt therapy to go over the patient’s difficult past. We can prepare a development plan, if the patient benefits from gestalt therapy. EXERCISE: Read books about topics resembling what you have experienced. PLAN: Trial gestalt session.

Patient history revealed that the patient had her uterus removed, which helped a little, but did not resolve the problem. At this stage, it is more difficult to process the patient due to the fact that the medical profession has burdened her with yet another trauma to process. Final developments of the patient will hopefully be gained during the gestalt therapy. Although the patient was found to have very little inner strength and courage to clear up and thus eliminate the pain, we must not give up on her beforehand.

## DISCUSSION

According to the holistic medical theory[5,12], physical pains are often existential pains that the patient will not assume responsibility for. This perspective can be sometimes difficult to understand for a person who has been educated within the biomedical paradigm and does not acknowledge the depth of existence, the nature of the human wholeness, and the causal nature of consciousness[17,18,19,20,21,22,23,24,28].

It is very important to rule out any serious and life-threatening diseases when a patient presents with complaints such as stomach pain, but when all medical inquiries and examinations have been exhausted without any results, treating the pain is often a simple procedure using the holistic medical toolbox[3,29]. In some cases, when the pain is in the region of the pelvis and a pelvic examination is to be carried out, we find it of value to use the holistic approach to the pelvic examination[30]. This is extremely crucial in a case where the patient is scared and sensitive. If the complaints of the patient are related to social problems[31], these must be resolved. If the patient is a child, the parents might be involved in the process of healing[32].

It is quite surprising that what can be considered one of the toughest challenges in the biomedical clinic can sometimes be one of the simplest problems to deal with in the holistic clinic. In our experience, pains in the internal organs of unknown origin are almost always caused by repressed emotions, giving the psychosomatic reaction. Using the holistic medical toolbox, the patient is motivated towards personal development and can often be cured of the pains when he or she assumes responsibility for the repressed feelings. The holistic process theory of healing[10,11,12,13] and the related quality of life theories[14,15,16] state that the return to the natural and pain-free state of being is possible whenever the person gets the resources needed for existential healing. We believe, and our clinical experience has constantly verified, that the resources needed are “holding” or genuine care in the dimensions of awareness, respect, care, acknowledgment, and acceptance with emotional support and processing in the dimensions of emotion, understanding, and abandoning negative attitudes and beliefs. The precondition for the holistic healing to take place is trust between the physician and the patient, which seems to be the biggest challenge of holistic medicine, especially when dealing with the patient in pain.

## ACKNOWLEDGMENTS

This study was supported by grants from IMK Almene Fond. The quality of life research was approved by the Copenhagen Scientific Ethical Committee under number (KF)V.100.2123/91.

## BIOSKETCHES

**Søren Ventegodt, MD,** is the director of the Quality of Life Research Center in Copenhagen, Denmark. He is also responsible for a Research Clinic for Holistic Medicine in Copenhagen and is a popular speaker throughout Scandinavia. He has published numerous scientific or popular articles and a number of books on holistic medicine, quality of life, and quality of working life. His most important scientific contributions are the comprehensive SEQOL questionnaire, the very short QoL5 questionnaire, the integrated QOL theory, the holistic process theory, the life mission theory, and the ongoing Danish Quality of Life Research Survey, 1991–94 in cooperation with the University Hospital of Copenhagen and the late professor of pediatrics, Bengt Zachau-Christiansen, MD, PhD. E-mail: ventegodt@livskvalitet.org. Website: http://www.livskvalitet.org

**Joav Merrick, MD, DMSc,** is professor of child health and human development affiliated with the Zusman Child Development Center, Division of Pediatrics and Community Health at the Ben Gurion University, Beer-Sheva, Israel; the medical director of the Division for Mental Retardation, Ministry of Social Affairs, Jerusalem; and founder and director of the National Institute of Child Health and Human Development. He has numerous publications in the field of child health and human development, rehabilitation, intellectual disability, disability, health, welfare, abuse, advocacy, quality of life and prevention. Dr. Merrick received the Peter Sabroe Child Award for outstanding work on behalf of Danish Children in 1985 and the International LEGO-Prize ("The Children's Nobel Prize") for an extraordinary contribution towards improvement in child welfare and well being in 1987. E-mail: jmerrick@internet-zahav.net. Website: www.nichd-israel.com
